# Supplementary material for: The Evolution and Characterization of the RNA Interference Pathways in Lophotrochozoa
Source: Genome Biol Evol. 2024 May 7;16(5):evae098. doi: 10.1093/gbe/evae098 (PMC11114477; doi:10.1093/gbe/evae098)
Supplement: evae098_Supplementary_Data [file evae098_supplementary_data.zip › Supplementary_materials.zip!/selected_miRNAs/NoGe_selected_miRNAs/known_miRNA_families_pdf_files/NMRB01000494.1_Notospermus_geniculatus_strain_Ushimado_scaffold494,_whole_genome_shotgun_sequence_14538.pdf]

Provisional ID	:	NMRB01000494.1_Notospermus_geniculatus_strain_Ushimado_scaffold494,_whole_genome_shotgun_sequence_14538
Score total	:	82762.4
Score for star read(s)	:	3.9
Score for read counts	:	82754.4
Score for mfe	:	1.1
Score for randfold	:	0
Score for cons. seed	:	3
Total read count	:	162331
Mature read count	:	162183
Loop read count	:	0
Star read count	:	148

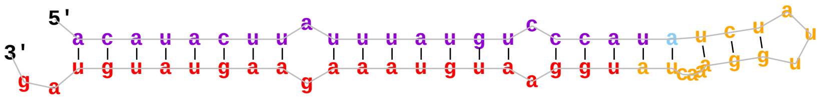

Genome coordinates: NMRB01000494.1 98198-98256

pre-miRNA sequence:
ACAUACUUAUUUAUGUCCCAUACAUACUUAUUUAUGUCCCAUAUCUAUUGGAAACUAUGGAAUGUAAAGAAGUAUGUAG

Mature sequence: UGGAAUGUAAAGAAGUAUGUAG

Aligned mature sequence of the *N. geniculatus* pre-miRNA with the top 5 hits from the BLAST search against the pre-miRNA mirGeneDB set:

```
                                     *********************
    Nge_Mir-1-P3                     uggaauguaaagaaguauguag
    Loc-Mir-1-P1_3p                  uggaauguaaagaaguauguau
    Tni-Mir-1-P1_3p                  uggaauguaaagaaguauguau
    Aca-Mir-1-P3_3p                  uggaauguaaagaaguauguau
    Gja-Mir-1-P3_3p                  uggaauguaaagaaguauguau
    Pbv-Mir-1-P3_3p                  uggaauguaaagaaguauguau
```
